# Paired-ear tympanometric pressure patterns and hearing recovery in unilateral sudden sensorineural hearing loss: a retrospective cohort study

**DOI:** 10.3389/fneur.2026.1982019

**Published:** 2026-10-16

**Authors:** Tianyu Zhang, Yaxue Xie, Yingxuan Li, Haibo Zhang, Hanzhai Tian, Ziye Zhu, Zhiyu Yang, Zhenlin Wang, Jiali Duan, Tianyang Zhang

**Affiliations:** 1 Department of Otolaryngology, Xiongan Xuanwu Hospital, Xiongan, China; 2 Department of Otolaryngology, Xiong'an Health Management Center, Peking University People's Hospital, Xiongan, China; 3 Department of Otolaryngology, Xuanwu Hospital Capital Medical University, Beijing, China; 4 Department of Otolaryngology, Rongcheng Traditional Chinese Medicine Hospital, Xiongan, China

**Keywords:** hearing recovery, interaural pressure asymmetry, paired-ear phenotyping, sudden sensorineural hearing loss, tympanometric peak pressure

## Abstract

**Background:**

Sudden sensorineural hearing loss (SSNHL) remains difficult to phenotype and predict. Tympanometry is routinely performed, yet its quantitative pressure information is largely discarded once conductive hearing loss is excluded. We used a paired-ear strategy to compare middle-ear pressure between the affected and contralateral ears in unilateral SSNHL.

**Methods:**

We retrospectively studied 203 adults with unilateral SSNHL and bilateral tympanometric peak pressure (TPP) measurements. Rather than relying on a single abnormality threshold, we integrated signed interaural differences, absolute pressure asymmetry, laterality-aware pressure phenotypes, and threshold-sensitivity analyses. Among patients with follow-up audiometry, hearing recovery was quantified as both continuous hearing gain and clinically meaningful ≥15-dB improvement, and was further examined in relation to categorical and continuous TPP phenotypes.

**Results:**

Paired-ear analysis showed no significant pressure shift in the affected ear compared with the contralateral ear (*p* = 0.410), indicating no consistent affected-ear pressure abnormality at the population level. Laterality-aware classification identified affected-ear-only negative pressure in 14 patients (6.9%) and contralateral-ear-only negative pressure in 11 patients (5.4%). This subgroup would be largely invisible using conventional single-ear classification. Across complementary binary, continuous, and multivariable analyses, tympanometric pressure showed no robust relationship with short-term hearing recovery, and this conclusion remained stable across alternative pressure thresholds.

**Conclusion:**

Unilateral SSNHL is not characterized by a universal middle-ear pressure abnormality. Paired-ear assessment can identify individual differences in middle-ear pressure that may be overlooked by single-ear classification. However, baseline TPP was not clearly associated with short-term hearing recovery in this cohort.

## 1 Introduction

Sudden sensorineural hearing loss can change hearing within hours, yet its clinical course remains remarkably unpredictable. Current research largely focuses on cochlear injury, inflammation, vascular dysfunction, and systemic risk factors (1, 2). Prognostic models also emphasize age, baseline hearing, vertigo, treatment delay, and audiogram pattern (3–7). These variables are useful, but they do not explain why patients with similar presentations recover so differently. Meanwhile, one routine measurement is usually collected, checked, and then ignored. That measurement is tympanometric peak pressure, or TPP. TPP is commonly used to exclude conductive disease, not to phenotype SSNHL. Once conductive pathology is excluded, its numerical value often disappears from subsequent clinical reasoning. This may discard physiological information that is already available at no additional testing cost.

Middle-ear pressure is not a simple “normal” or “abnormal” signal. It reflects Eustachian-tube ventilation, mucosal gas exchange, and the mechanical state of the tympanic membrane and ossicular chain (8, 9). Recent studies also show that resting tympanometry may miss dynamic Eustachian-tube dysfunction (9, 10). In SSNHL, Eustachian-tube function may even influence intratympanic steroid delivery and treatment response (11–13). Wideband tympanometry further shows that middle-ear mechanics vary across individuals and cannot be captured by one categorical label (13, 14). Yet most SSNHL studies still treat tympanometry as a screening step. They rarely preserve bilateral numeric pressure values. They also rarely ask whether the affected ear behaves differently from the patient’s own contralateral ear. Consequently, potentially informative interaural relationships remain largely invisible. This is the gap our study targets.

We used the contralateral ear as an internal reference for within-patient comparison. This design removes much of the between-patient noise that complicates absolute TPP comparisons. Both ears share age, systemic condition, testing environment, and most short-term physiological influences. We therefore compare pressure within the same person, rather than across unrelated patients. We also avoid reducing TPP to a single threshold. First, we calculate the signed interaural pressure difference. Second, we quantify the absolute magnitude of pressure asymmetry. Third, we classify both ears into four laterality-aware pressure patterns. Finally, we repeat the analysis across several negative-pressure thresholds. This framework separates direction, magnitude, laterality, and threshold stability. This approach allows TPP to be assessed according to direction, magnitude, laterality, and threshold. No single conventional tympanometric label provides all four dimensions (11, 15).

Using this paired-ear framework, we ask three clinically simple but previously overlooked questions. Does unilateral SSNHL produce a consistent pressure shift on the affected side? Do some patients show marked interaural pressure differences despite overall symmetry at the population level? Does interaural pressure discordance carry prognostic information beyond a binary abnormality? Our approach does not assume that negative pressure causes SSNHL. Instead, it tests whether routine tympanometry can identify different interaural pressure patterns. The method is inexpensive, scalable, and immediately compatible with real-world clinical records. It also requires no new device, biomarker assay, or imaging sequence. This paired-ear approach allows bilateral measurements to be interpreted within the same patient rather than in isolation.

## 2 Methods

### 2.1 Study design and setting

This retrospective cohort study included patients with unilateral sudden sensorineural hearing loss (SSNHL) evaluated at Xiong’an Xuanwu Hospital between February 2024 and January 2026. A paired-ear approach was adopted to compare tympanometric characteristics between the affected and contralateral ears within each patient. Clinical and audiological data, including audiometric and tympanometric measurements, were extracted from electronic medical records and audiology databases. The study was conducted in accordance with the Declaration of Helsinki and reported following the Strengthening the Reporting of Observational Studies in Epidemiology (STROBE) guidelines. The study protocol was approved by the Ethics Committee of Xiong’an Xuanwu Hospital (Approval number: XA Clinical Trial Approval [KS2024] No. 010-002). The requirement for informed consent was waived because of the retrospective nature of the study and the use of de-identified clinical data.

### 2.2 Patient selection and eligibility criteria

SSNHL was defined as a sensorineural hearing loss of ≥30 dB across at least three consecutive frequencies occurring within 72 h. The diagnosis was established after clinical and audiological evaluation. Patients with an identifiable cause of sudden hearing loss based on medical history, otologic examination, audiological assessment, and clinically indicated imaging or laboratory findings were excluded. The inclusion criteria were as follows: (1) age ≥18 years; (2) diagnosis of unilateral SSNHL with the affected side clearly documented; and (3) completion of bilateral tympanometric peak pressure (TPP) measurements at the initial evaluation.

The exclusion criteria were as follows: (1) bilateral sudden hearing loss; (2) conductive or mixed hearing loss; (3) active middle-ear effusion or acute otitis media; (4) tympanic membrane perforation; (5) previous ear surgery that could affect tympanometric measurements; and (6) missing numeric TPP data for either ear.

### 2.3 Clinical and audiological assessment

Clinical characteristics included age, sex, affected side, tinnitus, vertigo, diabetes, hypertension, time from symptom onset to treatment, and treatment with systemic or intratympanic corticosteroids. Pure-tone audiometry results from the initial evaluation and follow-up assessments were obtained from the audiological records. Pure-tone average (PTA) was calculated as the mean air-conduction hearing threshold at 0.5, 1, 2, and 4 kHz. Hearing recovery was evaluated using both continuous and categorical measures. Hearing gain was calculated as the initial PTA minus the follow-up PTA, with positive values indicating improvement. A hearing gain of ≥15 dB was considered clinically meaningful recovery, consistent with the magnitude of improvement used in established SSNHL recovery classifications. Hearing gain was also analyzed continuously to avoid relying on this cutoff alone (16). Analyses of hearing recovery were restricted to patients who completed follow-up audiometry.

Tympanometric data from the initial evaluation were extracted from the audiological records. TPP measurements were obtained at the initial evaluation after symptom onset and before the initiation of systemic or intratympanic corticosteroid treatment. Tympanometry was performed using a GSI TympStar middle-ear analyzer (Grason-Stadler, Eden Prairie, MN, USA) with a 226-Hz probe tone. The device was calibrated according to the manufacturer’s recommendations and routine audiology laboratory procedures. Measurements were obtained with patients seated in a quiet testing environment after otoscopic examination and confirmation of an adequate probe seal. Both ears were tested using the same standardized protocol during the initial evaluation, and TPP was automatically determined by the device from the pressure at maximum tympanic-membrane admittance. Numeric tympanometric peak pressure (TPP), expressed in daPa, was recorded for both ears. A TPP ≤ −100 daPa was used to define negative middle-ear pressure. Additional thresholds of −50, −75, −125, and −150 daPa were examined in sensitivity analyses to assess the robustness of pressure classification.

### 2.4 Interaural pressure differences and bilateral pressure classification

Using −100 daPa as the primary threshold for negative middle-ear pressure, patients were classified into four bilateral pressure patterns according to the TPP values of the affected and contralateral ears: (1) concordant non-negative, when TPP was > − 100 daPa in both ears; (2) affected-only negative, when TPP was ≤ − 100 daPa in the affected ear but > − 100 daPa in the contralateral ear; (3) contralateral-only negative, when TPP was > − 100 daPa in the affected ear but ≤ − 100 daPa in the contralateral ear; and (4) bilateral negative, when TPP was ≤ − 100 daPa in both ears.

### 2.5 Statistical analysis

Continuous variables were summarized as mean ± standard deviation (SD) or median with interquartile range (IQR), according to their distributions, and categorical variables as number and percentage. Paired TPP values from the affected and contralateral ears were compared using the Wilcoxon signed-rank test, and their correlation was assessed using Spearman’s rank correlation coefficient. The prevalence of negative middle-ear pressure was reported with 95% confidence intervals (CIs), and differences in negative-pressure status between paired ears were evaluated using the exact McNemar test. Differences between paired TPP measurements were further evaluated using Bland–Altman analysis. Among patients with follow-up audiometry, hearing gain was compared according to affected-ear negative-pressure status using the Mann–Whitney U test, while the proportion achieving ≥15-dB recovery was compared using Fisher’s exact test. Multivariable linear regression with heteroscedasticity-robust HC3 standard errors was used to examine the association between affected-ear negative pressure and hearing gain. Potential confounders were identified *a priori* based on established prognostic factors for hearing recovery in SSNHL and their clinical relevance to the exposure–outcome relationship. Age, baseline PTA, treatment delay, and vertigo were selected based on previous evidence rather than statistical significance in the present dataset.

Given the small number of patients with affected-ear negative pressure in the follow-up cohort, model stability was further assessed using bootstrap resampling. The adjusted regression model was repeated in 1,000 bootstrap samples to evaluate the stability of the estimated association and its confidence interval.

Continuous pressure–recovery relationships were examined using signed ΔTPP and absolute interaural asymmetry. Spearman correlation and locally weighted scatterplot smoothing (LOWESS) were used to characterize unadjusted relationships. An exploratory multivariable model incorporating signed ΔTPP and absolute interaural asymmetry was used to examine the relative contributions of pressure direction and magnitude, with both measures scaled per 10 daPa. Sensitivity analyses repeated the negative-pressure models using TPP thresholds of −50, −75, −125, and −150 daPa. The −100 daPa cutoff was prespecified as the primary threshold. Alternative thresholds were examined only as sensitivity analyses to assess whether the findings depended on the selected cutoff. Because these thresholds were nested and highly correlated and did not represent independent primary hypothesis tests, no formal adjustment for multiple comparisons was applied. Findings from these exploratory analyses were interpreted cautiously. These threshold analyses and the direction–magnitude model were considered exploratory. All tests were two-sided, with *p* < 0.05 considered statistically significant. Statistical analyses were performed using Python with SciPy and statsmodels. Missing data were not imputed. All 203 included patients had complete bilateral TPP measurements, as required by the eligibility criteria. Analyses involving other variables were performed using available cases. Hearing-recovery analyses were restricted to patients with available follow-up audiometry.

## 3 Results

### 3.1 Clinical and audiological characteristics

During the study period, 286 patients with SSNHL were screened for eligibility, of whom 83 were excluded according to the predefined criteria (Figure 1). The remaining 203 patients with unilateral SSNHL and complete bilateral TPP measurements were included in the paired-ear analyses. Follow-up audiometry was available for 162 patients, who were included in the hearing-recovery analyses. Their clinical and audiological characteristics were obtained from existing medical and audiology records and are summarized in Table 1. The study population was middle-aged overall, with a relatively balanced distribution of SSNHL between the right and left sides. Tinnitus was common, whereas vertigo and major comorbidities were less frequent. At the initial evaluation, PTA was substantially higher on the SSNHL side than on the contralateral side (64.2 ± 19.4 vs. 20.1 ± 9.1 dB). This pronounced within-patient difference reflects the unilateral nature of the study population and provides the clinical basis for comparing tympanometric pressure between the two ears within the same patient. Most patients received systemic corticosteroid treatment, and the median time from symptom onset to treatment was 4.0 days. Bilateral TPP measurements were recorded for all 203 patients and were used for the paired-ear pressure analyses. Follow-up audiometric data were available for 162 patients, who were subsequently included in the hearing recovery analyses. Baseline clinical, audiological, and tympanometric characteristics were similar between patients with and without follow-up audiometry, with no significant between-group differences (Supplementary Table S1).

**Figure 1:**
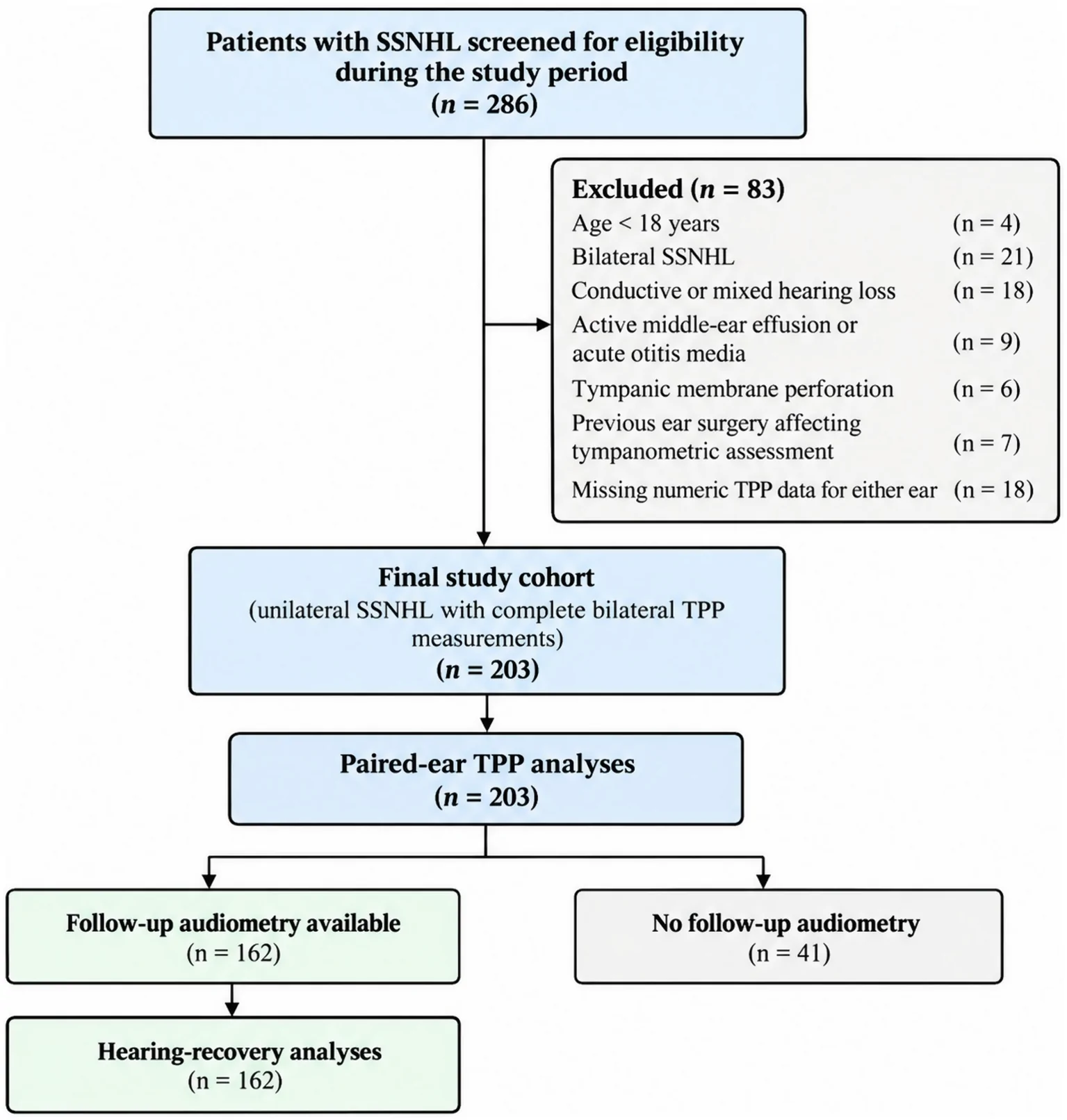
Flow diagram of patient screening, eligibility assessment, and inclusion in the study analyses. TPP, tympanometric peak pressure; SSNHL, sudden sensorineural hearing loss.

**Table 1:** Clinical and audiological characteristics of the study population.

Characteristic	Value	Missing, *n* (%)
Age, years	52.5 ± 13.9	0 (0%)
Male sex	120 (59.1%)	0 (0%)
Right-sided SSNHL	110 (54.2%)	0 (0%)
Tinnitus	172 (86.0%)	3 (1.5%)
Vertigo	49 (24.4%)	2 (1.0%)
Diabetes	25 (12.4%)	2 (1.0%)
Hypertension	46 (22.9%)	2 (1.0%)
Initial PTA, SSNHL side, dB	64.2 ± 19.4	0 (0%)
Contralateral-ear PTA, dB	20.1 ± 9.1	0 (0%)
Onset-to-treatment interval, days	4.0 (3.0–5.0)	4 (2.0%)
Systemic corticosteroid treatment	191 (94.6%)	1 (0.5%)
Intratympanic corticosteroid treatment	75 (37.1%)	1 (0.5%)
Follow-up audiometry	162 (79.8%)	41 (20.2%)
Follow-up interval, days	23.0 (17.0–29.8)	41 (20.2%)

Values are presented as mean ± SD, median (IQR), or *n* (%). Percentages for variables with missing data were calculated using the number of patients with available data as the denominator. Missing data were not imputed. The follow-up interval was calculated among the 162 patients with available follow-up audiometry. PTA, pure-tone average; SSNHL, sudden sensorineural hearing loss.

### 3.2 Interaural relationship and differences in tympanometric peak pressure

To determine whether unilateral SSNHL is associated with a disease-side–specific alteration in middle-ear pressure, we compared TPP between the affected and contralateral ears using a within-patient paired approach (Figures 2A,B). TPP distributions were similar between ears, with median values of −9.1 daPa in the affected ear and −8.1 daPa in the contralateral ear. The median signed interaural difference was small (−1.2 daPa) and did not indicate a systematic shift toward lower pressure on the affected side (*p* = 0.410). The median absolute interaural asymmetry was 11.9 daPa, although a limited number of patients showed substantially larger between-ear differences. Overall, these findings indicate that middle-ear pressure is largely symmetric at the cohort level and that unilateral SSNHL is not accompanied by a consistent affected-ear pressure deviation. Nevertheless, the presence of marked asymmetry in a subset of patients supports further characterization of individual bilateral pressure phenotypes.

**Figure 2:**
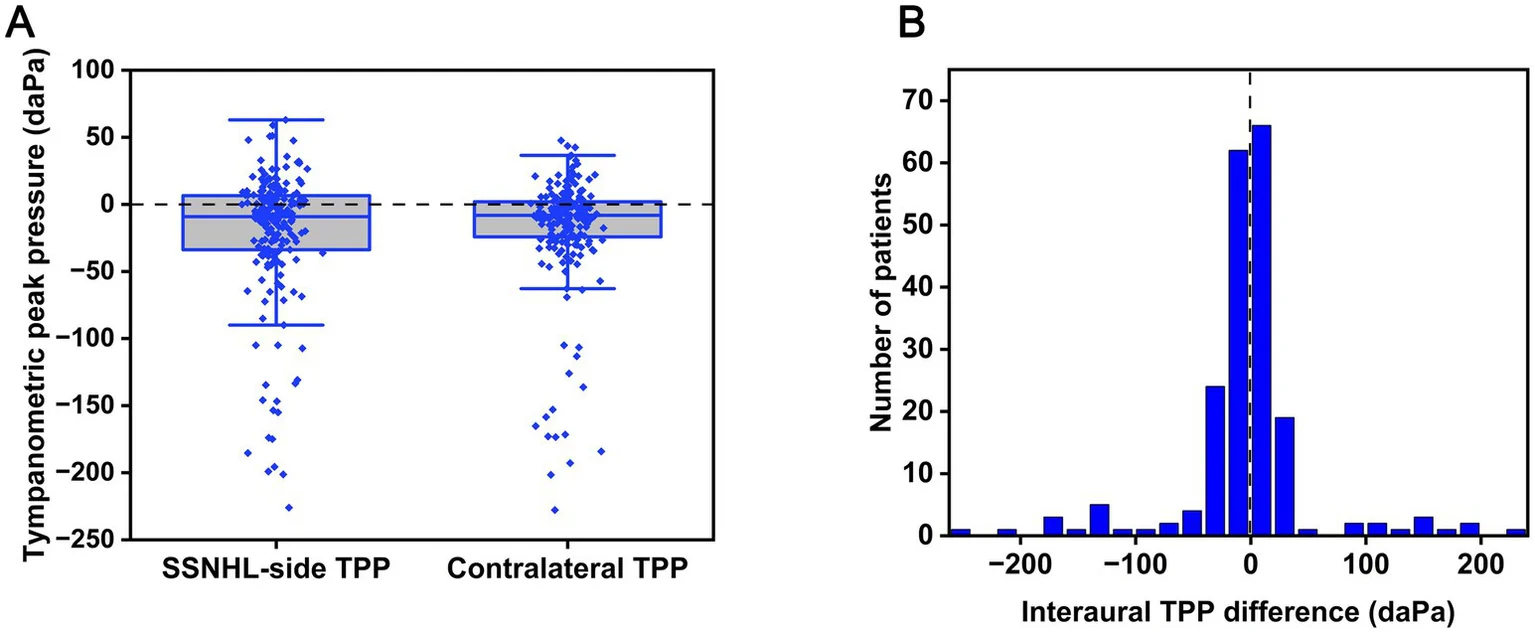
Tympanometric peak pressure in the affected and contralateral ears. **(A)** TPP values in the affected and contralateral ears. **(B)** Signed interaural TPP difference, calculated as affected-ear TPP minus contralateral-ear TPP.

### 3.3 Distribution of bilateral tympanometric pressure patterns

To characterize how negative middle-ear pressure was distributed between ears, patients were classified into four bilateral TPP phenotypes using −100 daPa as the predefined threshold (Table 2). Most patients had non-negative pressure in both ears (174/203, 85.7%). Among patients with negative pressure, 14 (6.9%) showed negative pressure only in the affected ear, 11 (5.4%) only in the contralateral ear, and 4 (2.0%) in both ears. As illustrated in Figure 3A, most paired TPP measurements were located within the bilateral non-negative region, whereas smaller subsets occupied the affected-only, contralateral-only, or bilateral-negative regions. Figure 3B further shows the relative frequency of these four phenotypes across the cohort. Overall, negative pressure was uncommon and was not confined to the SSNHL side, indicating heterogeneous bilateral pressure patterns and supporting assessment of both ears when characterizing tympanometric pressure in unilateral SSNHL.

**Table 2:** Interaural tympanometric peak pressure characteristics in patients with unilateral SSNHL.

Pressure feature	Value	Statistical note
Affected-ear TPP, daPa	−9.1 (−33.7 to 6.3)	—
Contralateral-ear TPP, daPa	−8.1 (−24.1 to 1.4)	—
Signed ΔTPP, daPa	−1.2 (−14.1 to 10.3)	*p* = 0.410
Absolute TPP asymmetry, daPa	11.9 (5.2 to 27.7)	—
Affected-ear negative pressure (TPP ≤ −100 daPa)	18 (8.9%)	95% CI, 5.7–13.6%
Contralateral-ear negative	15 (7.4%)	*p* = 0.690
Bilateral non-negative	174 (85.7%)	—
Affected-ear-only negative	14 (6.9%)	—
Contralateral-ear-only negative	11 (5.4%)	—
Bilateral negative	4 (2.0%)	—

TPP indicates tympanometric peak pressure; ΔTPP = affected-ear TPP minus contralateral-ear TPP.

**Figure 3:**
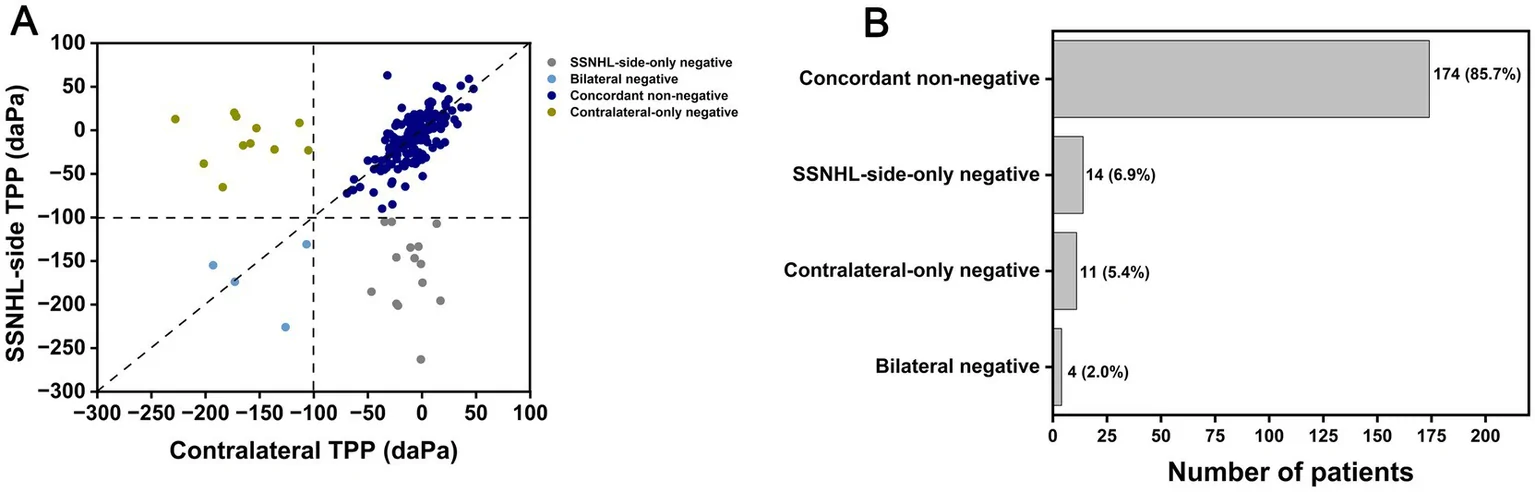
Bilateral tympanometric pressure phenotypes in unilateral SSNHL. **(A)** Paired affected-ear and contralateral-ear TPP values classified according to the −100 daPa threshold. Dashed lines indicate the negative-pressure cutoff, and the diagonal line indicates equal TPP in both ears. **(B)** Distribution of the four bilateral pressure phenotypes in the study cohort.

### 3.4 Continuous distribution of interaural TPP asymmetry

To assess interaural pressure differences beyond a binary negative-pressure classification, we examined absolute TPP asymmetry as a continuous measure. Across the cohort, the median absolute difference between the affected and contralateral ears was 11.9 daPa (IQR, 5.2–27.7 daPa). As shown in Figure 4, most patients had relatively small between-ear differences, whereas a smaller proportion showed substantially greater pressure asymmetry. The distribution was therefore concentrated at lower values with a broader upper range, indicating that marked interaural discordance was limited to a subset of patients. These findings show that bilateral TPP values were generally similar in most patients, while the degree of asymmetry varied considerably across individuals.

**Figure 4:**
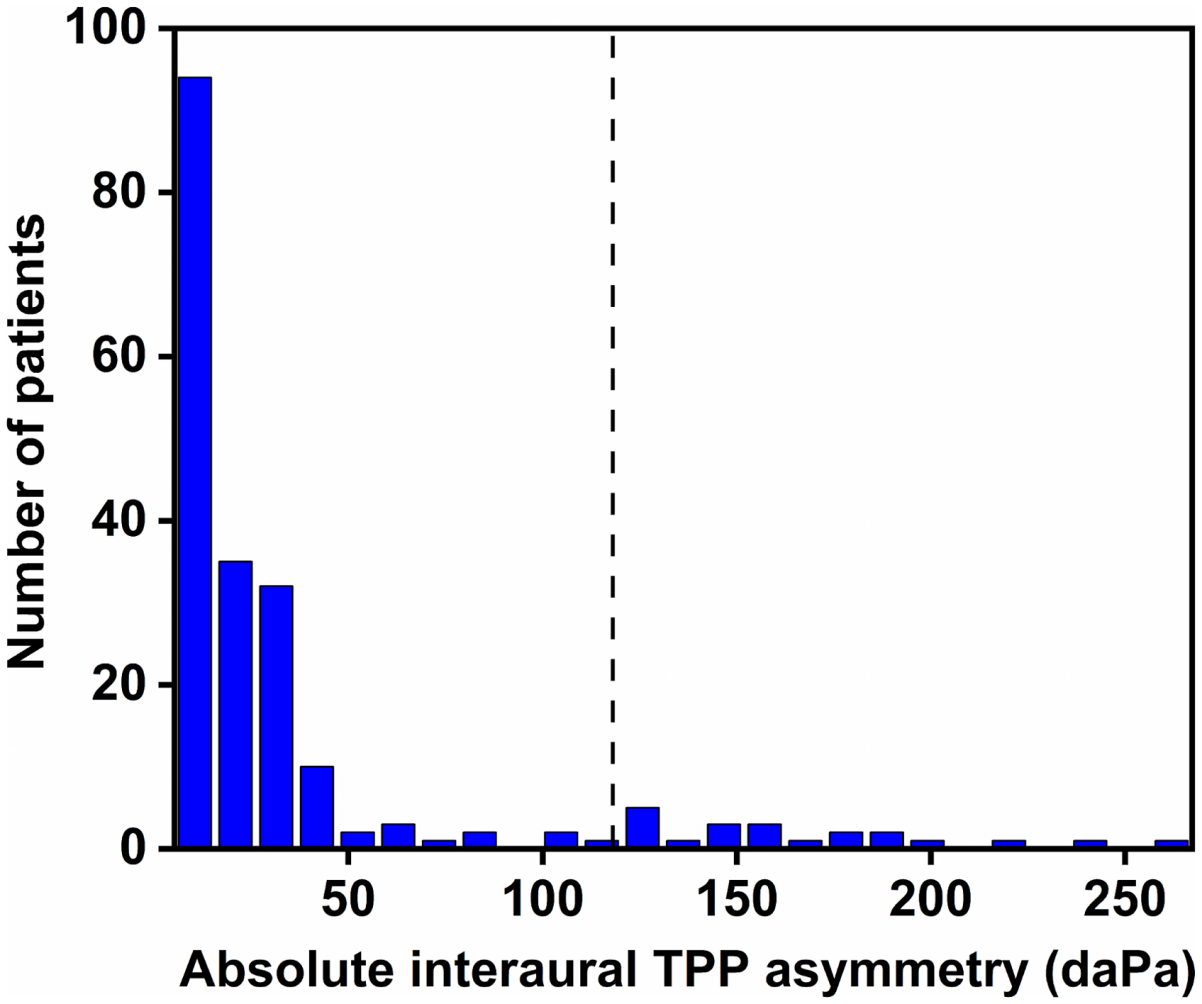
Distribution of absolute interaural tympanometric pressure asymmetry.

### 3.5 Sensitivity of bilateral pressure classification to alternative TPP thresholds

To examine whether bilateral pressure phenotypes were sensitive to the definition of negative middle-ear pressure, we repeated the classification using TPP thresholds ranging from −50 to −150 daPa. As shown in Figure 5A, the prevalence of negative-pressure phenotypes decreased progressively as the threshold became more stringent, reflecting the smaller number of patients meeting criteria for more severe negative pressure. Despite this change in prevalence, the overall pattern of bilateral pressure classification remained similar across thresholds. Figure 5B shows that affected-ear-only negative pressure was more frequent than contralateral-ear-only negative pressure at most cutoff values, although the difference between these two phenotypes became smaller at the most stringent threshold. Overall, the relative distribution of pressure phenotypes was broadly preserved across different definitions of negative pressure, suggesting that the observed bilateral pattern was not dependent on a single cutoff value.

**Figure 5:**
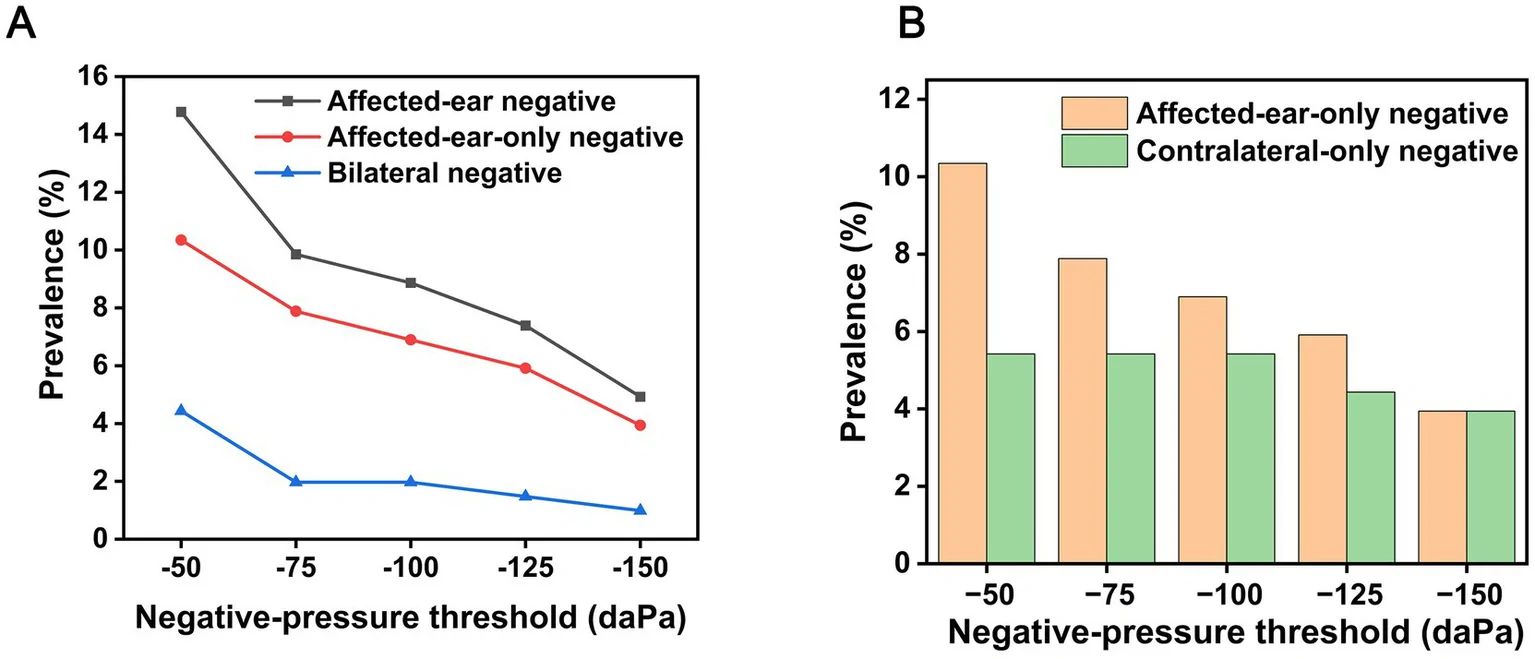
Tympanometric pressure phenotypes across alternative negative-pressure thresholds. **(A)** Prevalence of SSNHL-side negative, SSNHL-side-only negative, and bilateral negative pressure across thresholds from −50 to −150 daPa. **(B)** Prevalence of SSNHL-side-only and contralateral-only negative pressure across the same thresholds.

### 3.6 Association between affected-ear negative pressure and short-term hearing recovery

To assess whether affected-ear negative middle-ear pressure was associated with short-term hearing recovery, 162 patients with follow-up audiometry were grouped according to baseline TPP status using the −100 daPa threshold (Table 3). Mean hearing gain was similar in patients with and without negative pressure (19.8 vs. 23.2 dB; *p* = 0.648). Likewise, clinically meaningful recovery of ≥15 dB occurred in 71.4 and 70.3% of patients, respectively. The substantial overlap in hearing-gain distributions between groups is shown in Figure 6A, while Figure 6B demonstrates comparable recovery proportions. After adjustment for baseline PTA, age, treatment delay, and vertigo, affected-ear negative pressure remained unassociated with hearing gain (adjusted *β*, −3.7 dB; 95% CI, −11.1 to 3.7; *p* = 0.326). These results indicate that baseline affected-ear negative pressure alone did not distinguish patients with better or worse short-term hearing recovery in this cohort. Given the small number of patients with affected-ear negative pressure (*n* = 14), the adjusted estimate was interpreted cautiously. Bootstrap analysis yielded a similar effect estimate and did not materially alter the inference, although the confidence interval remained wide, reflecting limited precision.

**Table 3:** Short-term hearing outcomes according to SSNHL-side TPP status.

Outcome	No negative pressure	Negative pressure	Comparison
Follow-up n	148	14	—
Baseline affected-ear PTA, dB	63.1	65.5	—
Hearing gain, dB, median (IQR)	23.2	19.8	*p* = 0.648
≥15-dB recovery	104/148 (70.3%)	10/14 (71.4%)	*p* = 1.000
Adjusted *β* for hearing gain, dB	Reference	−3.7 (−11.1 to 3.7)	*p* = 0.326

Analyses were restricted to patients with follow-up audiometry. Negative middle-ear pressure was defined as TPP ≤ − 100 daPa. Hearing gain was calculated as baseline PTA minus follow-up PTA, with positive values indicating improvement. Clinically meaningful recovery was defined as a hearing gain of ≥15 dB. The adjusted β was estimated using HC3-robust linear regression controlling for baseline PTA, age, treatment delay, and vertigo. PTA, pure-tone average; TPP, tympanometric peak pressure; CI, confidence interval.

**Figure 6:**
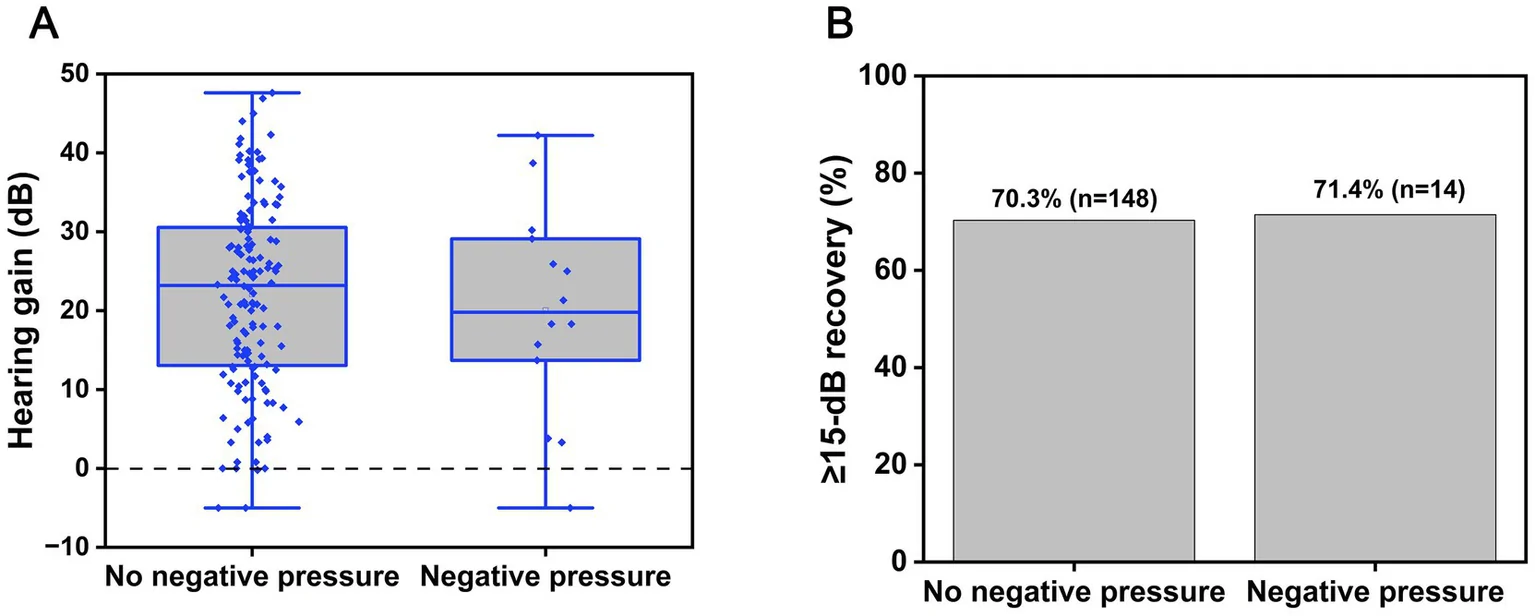
Short-term hearing recovery according to affected-ear middle-ear pressure status. **(A)** Distribution of hearing gain in patients with and without affected-ear negative pressure. **(B)** Proportion of patients achieving ≥15-dB hearing recovery in each group.

### 3.7 Continuous associations between interaural TPP differences and hearing recovery

To determine whether interaural pressure differences were related to short-term hearing recovery across their full range, we examined signed ΔTPP and absolute TPP asymmetry as continuous measures in patients with follow-up audiometry. As shown in Figure 7A, hearing gain remained broadly distributed across different signed ΔTPP values, and the LOWESS curve showed no clear increasing or decreasing pattern. A similar result was observed for absolute TPP asymmetry (Figure 7B), with hearing improvement showing no consistent change as the magnitude of the between-ear pressure difference increased. Although several patients had relatively large interaural pressure differences, these values were not accompanied by a distinct pattern of hearing gain. Overall, the unadjusted continuous analyses did not identify a clear relationship between either the direction or magnitude of interaural TPP differences and short-term hearing recovery, supporting further assessment using adjusted models.

**Figure 7:**
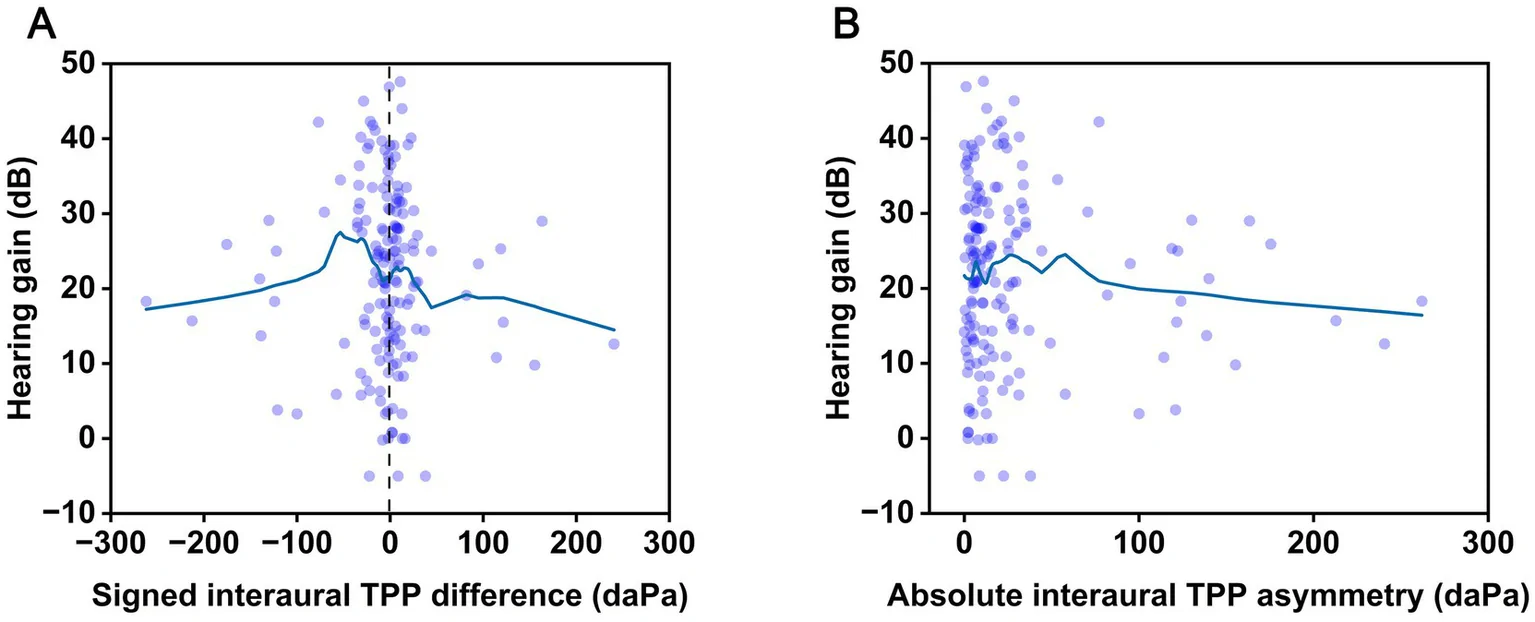
Continuous relationships between interaural tympanometric pressure differences and hearing gain. **(A)** Hearing gain according to signed ΔTPP, calculated as affected-ear TPP minus contralateral-ear TPP. **(B)** Hearing gain according to absolute interaural TPP asymmetry. Solid lines represent LOWESS-smoothed trends.

### 3.8 Adjusted analyses of interaural TPP measures and threshold sensitivity

To assess whether interaural TPP measures were independently associated with short-term hearing recovery, multivariable models were adjusted for baseline PTA, age, treatment delay, and vertigo (Figure 8A). Overall, the continuous pressure measures did not show a consistent independent relationship with hearing gain after adjustment. We then tested whether the association between affected-ear negative pressure and hearing gain depended on the selected TPP cutoff (Table 4; Figure 8B). Across thresholds from −50 to −125 daPa, adjusted effect estimates remained modest and confidence intervals included the null. At the most stringent threshold of −150 daPa, negative pressure was associated with lower hearing gain (adjusted β, −11.6 dB; 95% CI, −22.2 to −1.0; *p* = 0.032); however, this estimate was based on only six exposed patients. Overall, the findings were broadly stable across pressure definitions, with no consistent evidence that baseline TPP abnormalities independently predicted short-term hearing recovery.

**Figure 8:**
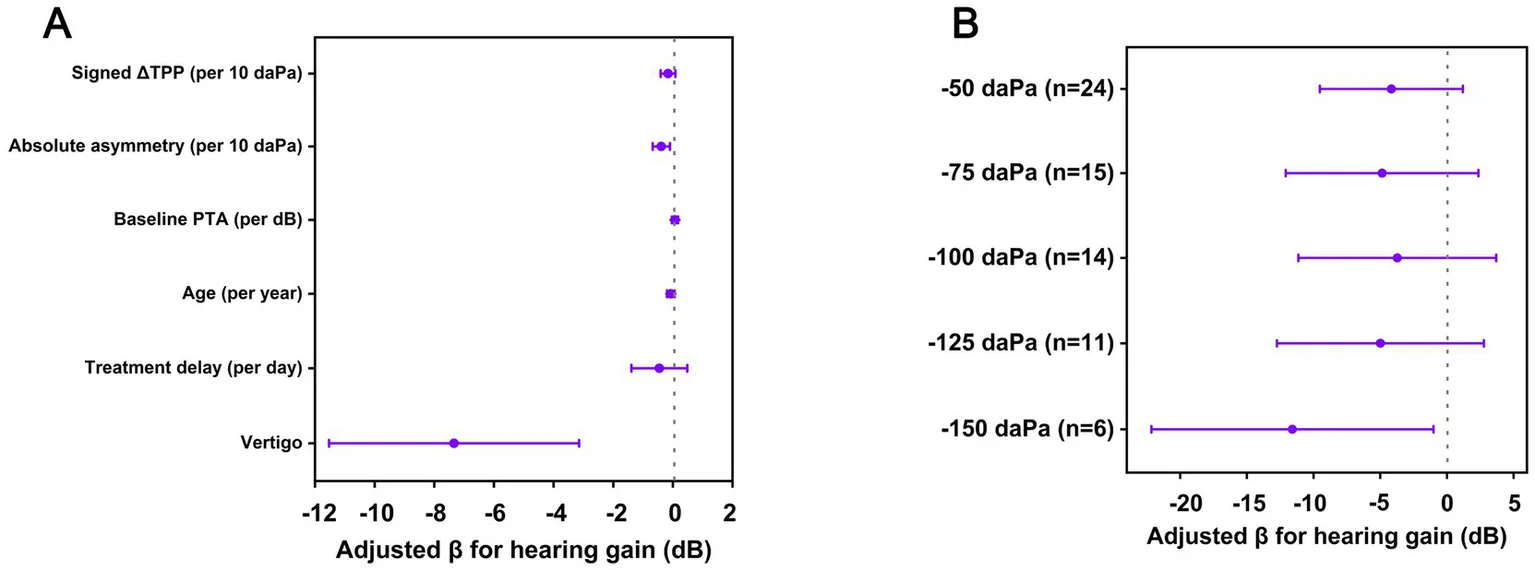
Adjusted associations between tympanometric pressure measures and hearing gain. **(A)** Multivariable associations of continuous interaural TPP measures with hearing gain after adjustment for baseline PTA, age, treatment delay, and vertigo. Points indicate adjusted β coefficients and error bars indicate 95% confidence intervals. **(B)** Adjusted associations between affected-ear negative pressure and hearing gain across TPP thresholds from −50 to −150 daPa.

**Table 4:** Sensitivity analysis of affected-ear negative-pressure thresholds and hearing gain.

Affected-ear threshold (daPa)	Follow-up negative *n*	Adjusted *β* (dB)	95% CI	*p*
−50	24	−4.2	−9.5 to 1.2	0.128
−75	15	−4.9	−12.1 to 2.4	0.188
−100	14	−3.7	−11.1 to 3.7	0.326
−125	11	−5.0	−12.7 to 2.8	0.208
−150	6	−11.6	−22.2 to −1.0	0.032

Each threshold was evaluated in a separate HC3-robust linear regression model adjusted for baseline PTA, age, treatment delay, and vertigo. Adjusted *β* values represent the difference in hearing gain associated with affected-ear negative pressure. The estimate at −150 daPa was based on six patients with negative pressure and should therefore be interpreted cautiously. PTA, pure-tone average; TPP, tympanometric peak pressure; CI, confidence interval.

## 4 Discussion

### 4.1 Principal findings and significance of the study

This study reassessed tympanometric pressure in unilateral SSNHL using a paired-ear approach. The principal finding was that SSNHL was not accompanied by a systematic affected-ear shift in TPP. Most patients had broadly symmetric, non-negative middle-ear pressure. A small proportion showed unilateral negative pressure, but the frequencies were similar in the affected and contralateral ears. Therefore, these findings do not support an affected-ear-specific pressure pattern at the group level. Importantly, affected-ear negative pressure was not associated with short-term hearing gain or clinically meaningful recovery, and this finding remained generally consistent across alternative pressure thresholds. These findings are clinically important because they clarify the role of TPP in SSNHL: baseline middle-ear pressure does not appear to provide reliable prognostic information for short-term hearing recovery. Rather, they define the clinical position of TPP in SSNHL: routine middle-ear pressure abnormalities should not be interpreted as a common feature of the disease or as an independent indicator of poor recovery. At the same time, bilateral quantitative assessment can identify uncommon pressure phenotypes that are lost when tympanometry is reduced to a binary normal/abnormal classification.

### 4.2 Interpretation in relation to current clinical evidence

Most established SSNHL prognostic models emphasize variables reflecting cochlear injury and overall disease severity, including baseline hearing loss, age, vertigo, treatment delay, and audiometric configuration (3, 17, 18). Middle-ear pressure has received substantially less attention because tympanometry is primarily used to exclude conductive pathology before confirming sensorineural hearing loss. Our findings support this clinical distinction. The absence of a consistent affected-ear pressure shift suggests that altered middle-ear ventilation is unlikely to represent a dominant pathophysiologic feature of SSNHL. This is consistent with the current understanding of SSNHL as a heterogeneous inner-ear disorder involving vascular, inflammatory, immune, infectious, or membrane-related mechanisms (1, 19, 20). Nevertheless, negative TPP is clinically meaningful in other contexts. Consensus criteria for Eustachian tube dysfunction recognize negative middle-ear pressure as objective evidence of impaired pressure regulation when accompanied by compatible symptoms or examination findings (21). Experimental studies have also demonstrated that negative pressure alters middle-ear sound transmission, wideband acoustic immittance, and otoacoustic-emission measurements (11, 22). The present results therefore refine, rather than dismiss, the relevance of TPP: middle-ear pressure may influence the acoustic and therapeutic environment of the ear, but it does not appear to characterize SSNHL itself in most patients.

### 4.3 Potential mechanisms and implications of interaural asymmetry

The paired-ear design also reveals an important distinction between absolute pressure and interaural discordance. Although population-level TPP was highly similar between ears, a minority of patients showed substantial asymmetry. The biological significance of these unilateral pressure patterns remains uncertain, particularly because affected-ear-only and contralateral-ear-only negative pressure occurred at similar frequencies. Such heterogeneity may reflect transient or unilateral Eustachian tube dysfunction, differences in middle-ear mechanics, recent upper-airway disease, or individual anatomic variation. Negative pressure increases middle-ear stiffness and alters transmission through the tympanic membrane–ossicular system, providing a plausible explanation for why pronounced pressure disturbances could modify audiologic measurements or the local conditions surrounding intratympanic treatment (11, 12). This distinction is particularly relevant because the prognostic signal was not consistently related to the direction of pressure change. The isolated association observed at the −150-daPa threshold may suggest that extreme pressure disturbance deserves further study, but the very small number of exposed patients precludes defining such a cutoff as prognostic. More broadly, these findings raise the possibility that the magnitude and persistence of interaural discordance may be biologically more informative than a single negative-pressure threshold. This concept requires prospective validation but provides a more precise framework for future investigation.

### 4.4 Clinical implications, limitations, and future directions

These findings do not support the use of a single TPP threshold to guide corticosteroid treatment or prognostic counseling. Although baseline TPP was not clearly associated with short-term hearing recovery, paired-ear assessment captured interaural pressure differences that would be overlooked by conventional single-ear classification. Retaining bilateral numeric TPP values may therefore facilitate more detailed characterization of interaural pressure patterns, although their clinical and prognostic relevance remains to be established. Future prospective studies should incorporate serial bilateral tympanometry, wideband acoustic immittance, Eustachian tube symptoms, otoscopic findings, and standardized hearing outcomes to determine whether these pressure patterns have clinical significance. Several limitations should be considered. First, the retrospective single-center design may have introduced selection bias and limits the generalizability of our findings to other clinical settings and populations. Second, information bias is possible because clinical characteristics, treatment information, and audiometric data were obtained retrospectively from medical records and were therefore dependent on the completeness and accuracy of routine documentation. Third, follow-up audiometry was unavailable for 41 patients, which may have introduced additional selection bias in the hearing-recovery analyses. Although baseline characteristics were similar between patients with and without follow-up, differences in unmeasured characteristics cannot be excluded; therefore, the direction and magnitude of any resulting selection bias remain uncertain. Fourth, potential Eustachian tube-related factors, including recent upper respiratory infection, allergic rhinitis, nasal obstruction, chronic rhinosinusitis, and ETD symptoms, were not systematically recorded and therefore could not be fully accounted for. These unmeasured factors may have contributed to the observed TPP differences and interaural asymmetry. Finally, the small number of patients with negative pressure in the follow-up cohort limited the precision and stability of the adjusted estimates.

## 5 Conclusion

This study examined whether routinely measured middle-ear pressure differs between ears and is related to hearing recovery in unilateral SSNHL. The findings suggest that SSNHL is not generally characterized by an affected-ear-specific pressure abnormality, and baseline negative pressure alone appears unlikely to provide reliable prognostic information for short-term hearing recovery. However, bilateral assessment identified a small subgroup with marked laterality-specific pressure abnormalities, indicating that interaural pressure patterns may capture heterogeneity that is overlooked by conventional single-ear classification. From a real-world clinical perspective, retaining bilateral numeric TPP values may offer a low-cost approach to refine phenotyping without adding tests or treatment burden. These data do not support modifying treatment solely according to baseline TPP, but they provide a practical framework for future risk-stratification studies. Prospective multicenter studies with complete clinical data and serial tympanometry are needed to determine whether persistent or dynamic pressure asymmetry has value for clinical decision-making.

## Data Availability

The raw data supporting the conclusions of this article will be made available by the authors, without undue reservation.
