# Supplementary material for: Paired-ear tympanometric pressure patterns and hearing recovery in unilateral sudden sensorineural hearing loss: a retrospective cohort study
Source: Front Neurol. 2026 Oct 16;17:1982019. doi: 10.3389/fneur.2026.1982019 (PMC13719301; doi:10.3389/fneur.2026.1982019)
Supplement: Supplementary file 1 [file Table_1.docx]

**Supplementary Table S1.** Comparison of baseline characteristics between patients with and without follow-up audiometry

| **Characteristic** | **With follow-up audiometry (n = 162)** | **Without follow-up audiometry (n = 41)** | **P value** |
| --- | --- | --- | --- |
| Age, years | 52.3 ± 13.8 | 53.2 ± 14.3 | 0.712 |
| Male sex, n (%) | 96 (59.3) | 24 (58.5) | 1.000 |
| Right-sided SSNHL, n (%) | 88 (54.3) | 22 (53.7) | 1.000 |
| Tinnitus, n (%) | 138 (85.2) | 34 (82.9) | 0.812 |
| Vertigo, n (%) | 36 (22.2) | 13 (31.7) | 0.231 |
| Diabetes, n (%) | 20 (12.3) | 5 (12.2) | 1.000 |
| Hypertension, n (%) | 36 (22.2) | 10 (24.4) | 0.838 |
| Initial PTA, SSNHL side, dB | 64.0 ± 19.2 | 65.1 ± 20.0 | 0.744 |
| Contralateral-ear PTA, dB | 20.0 ± 9.0 | 20.5 ± 9.4 | 0.756 |
| Affected-ear TPP, daPa | −9.0 (−33.5 to 6.5) | −9.5 (−34.0 to 5.0) | 0.884 |
| Contralateral-ear TPP, daPa | −8.0 (−24.0 to 1.5) | −8.5 (−24.5 to 1.0) | 0.921 |
| Signed ΔTPP, daPa | −1.1 (−14.0 to 10.2) | −1.5 (−14.5 to 10.5) | 0.907 |
| Absolute TPP asymmetry, daPa | 11.8 (5.1 to 27.5) | 12.2 (5.4 to 28.0) | 0.836 |
| Onset-to-treatment interval, days | 4.0 (3.0–5.0) | 4.5 (3.0–6.0) | 0.184 |
| Systemic corticosteroid treatment, n (%) | 153 (94.4) | 38 (92.7) | 0.721 |
| Intratympanic corticosteroid treatment, n (%) | 60 (37.0) | 15 (36.6) | 1.000 |
